# The role of MARCH9 in colorectal cancer progression

**DOI:** 10.3389/fonc.2022.906897

**Published:** 2022-09-16

**Authors:** Hua Liu, Biao Chen, Lian-Lin Liu, Lin Cong, Yong Cheng

**Affiliations:** ^1^ Key Laboratory of Ethnomedicine for Ministry of Education, Center on Translational Neuroscience, School of Pharmacy, Minzu University of China, Beijing, China; ^2^ Department of General Surgery, People’s Hospital of Tibet Autonomous Region, Lhasa, China; ^3^ Department of General Surgery, Peking Union Medical College Hospital, Chinese Academy of Medical Sciences & Peking Union Medical College, Beijing, China

**Keywords:** MARCH9, CYLD, p65, colorectal cancer, progression

## Abstract

Colorectal cancer (CRC) is the third most common cancer with a high global incidence and mortality. Mutated genes or dysregulated pathways responsible for CRC progression have been identified and employed as biomarkers for diagnosis and prognosis. In this study, a ubiquitination regulator, MARCH9, was shown to accelerate CRC progression both *in vitro* and *in vivo*. CRC samples from The Cancer Genome Atlas (TCGA) showed significantly upregulated MARCH9 expression by individual cancer stage, histological subtype, and nodal metastasis status. Knockdown of *MARCH9* inhibited, while *MARCH9* overexpression promoted, CRC cell proliferation and migration. Knockdown of *MARCH9* also induced CRC cell apoptosis and caused cell cycle arrest. Further investigation showed that MARCH9 promoted CRC progression by downregulating the expression of a deubiquitinase cylindromatosis (*CYLD*) gene and activating p65, a member of the nuclear factor-κB (NF-κB) protein family. Finally, *in vivo* xenograft studies confirmed that *MARCH9* knockdown suppressed tumor growth in nude mice. Thus, this study demonstrated that MARCH9 may be a novel and effective therapeutic target for CRC therapy.

## Introduction

According to the International Agency for Research on Cancer (IARC)’s GLOBOCAN 2020 database, there were more than 1.9 million new cases of colorectal cancer (CRC, including anus), with 935,000 deaths and 10% of all new cancer cases and deaths in 2020, respectively (1). In China, CRC is the fifth and fourth leading cause of cancer morbidity among men and women, respectively, the fifth leading cause of cancer mortality for both men and women (2). Current CRC treatment includes surgical therapy, postoperative chemotherapy, neoadjuvant radiotherapy and chemotherapy, maintenance therapy, immunotherapy, or targeted therapy according to each patient’s clinical situation and the known epidemiology in particular regions (3). CRC develops through a series of genetic, histological, and morphological changes that accumulate over time (4). Thus, it is necessary to define the potential mechanism of CRC tumorigenesis and progression.

Several studies have suggested that dysregulation of ubiquitin-mediated degradation may correlate with the occurrence and development of cancers (5). Membrane-associated RING-CH (MARCH) proteins were confirmed as ubiquitination regulators of major histocompatibility complex (MHC) proteins firstly (6). They include 11 family members, MARCH1 to MARCH 11, that play a role in immune regulation, dendritic cell (DC) maturation, antigen presentation, T cell development, thyroid hormone activity regulation, bile transport, endosomal trafficking, cell polarity, spermiogenesis, mitochondrial dynamics, protein quality control, and Toll-like receptor (TLR) signaling (7). Recent studies have found that MARCH protein expression correlates with cancer progression. MARCH1, MARCH2, MARCH3, and MARCH8 are associated with CRC (8–11), MARCH1, MARCH3, and MARCH6 are correlated with hepatocellular carcinoma (12–14), and MARCH1, MARCH5, MARCH7, and MARCH10 are associated with ovarian cancer (15–18).

MARCH9 is found in lysosomes and the trans-Golgi network (TGN) and is highly expressed in lymph tissue, lung tissue, DCs, T cells, and B cells (19–21). MARCH9 may participate in cellular immune regulation by downregulating MHC1, CD4, and ICAM-1 expression (22, 23). Luoto et al. used computational analysis to show that the CDK4-MARCH9 locus was expressed in the glioblastoma immune microenvironment and could potentially be used to facilitate patient stratification and improve personalized immunotherapy (24). Shen et al. reported that MARCH9 suppressed the progression of lung adenocarcinoma, and downregulation correlated with poor clinical outcomes (23). This manuscript explores a potential function and possible mechanism for MARCH9 in CRC.

## Materials and methods

### Clinical samples from the genome cancer atlas (TGCA)

CRC samples (n=471) and control samples (n=41) were downloaded from the TCGA-Colon Adenocarcinoma (COAD) database and MARCH9 expression was assessed by individual cancer stage (stage 1-stage 4), histological subtype (adenocarcinoma and mucinous adenocarcinoma), and nodal metastasis status (N0-N2).

### Cell culture and transfection

For cell lines, the human colonic cancer cell lines, HCT116 and RKO, were chosen and purchased from ATCC. Cells were cultured in Dulbecco’s modified Eagle’s medium (DMEM, Hyclone) that was supplemented with 10% fetal bovine serum (FBS, Hyclone) and 1% penicillin-streptomycin solution (Solarbio), and incubated in 5% CO_2_ (PHcbi, MCO-18AC). Lipofectamine RNA-iMax reagent (Invitrogen) was used for cell transfection. The sequences of si*MARCH9*#1 (5’-GCAGTGGAAGGTCCTAAATTA-3’), si*MARCH9*#2 (5’-GTCCAGATTGCTGCCATAGTT-3’) and siCtrl(5’-UUCUCCGAACGUGUCACGU-3’) were synthesized from GenePharma (Shanghai). The VigoFect repeat (Vigorous Biotechnology) was used for *MARCH9* overexpression.

### Quantitative real-time polymerase chain reaction (RT-qPCR)

The total RNAs of the HCT116 and RKO cells were isolated using Trizol Reagent. (Ambion, Life Technologies). ReverTra Ace qPCR RT Master Mix with gDNA Remover (Toyobo) was used for reverse transcription and 2* SYBR Green qPCR Master Mix (Low ROX) (Servicebio) was used for real-time PCR. The following primers were utilized: *MARCH9*-F: 5’-CTCCTCTGTCTACCGCATCTT-3’; *MARCH9*-R: 5’-TCTCCTCCTATGTCCTTGGTCT-3’; *GAPDH*-F: 5’-GACTCATGACCACAGTCCATGC-3’; and *GAPDH*-R: 5’-AGAGGCAGGGATGATGTTCTG-3’.

### Western blotting

Whole-cell lysates were prepared in RIPA lysis buffer (Beyotime) containing a protease cocktail (Beyotime). BCA Protein Assay Kit (Thermo) was used to evaluate total protein concentrations. Proteins were separated with 10% SDS-PAGE and transferred to a nitrocellulose membrane, and the membrane was blocked with 0.5% bovine serum albumin (BSA) for 1 h at 25°C. Anti-MARCH9 (proteintech, 1:1000), anti-CYLD (Abcam 1:1000), anti-p-p65 (1:1000), and anti-vinculin (ABclonal, 1:1000) antibodies were added to the membranes and incubated at 4°C overnight, separately. After washing three times with TBST, secondary antibodies were added and the membrane was incubated for 1 h at 25°C. IRDye@800CW reagent (LI-COR) and Odyssey CLx imager (LI-COR) were used to visualize the protein bands.

### Colony formation assay

Two CRC cell lines containing about 300 cells each were planted into 6 cm tissue culture plates and 4 mL DMEM was added. The cells were cultured for 3 weeks to form colonies. The cells were then fixed in 100% methanol for 20 min and stained with 0.1% crystal violet solution (Solarbio) for 10 min. The positive colonies were photographed and quantitated using a digital camera.

### Transwell migration assay

Transfected HCT116 and RKO cells were seeded in the upper chamber of a Transwell plate in DMEM containing 10% FBS. DMEM containing 20% FBS was added to the lower chamber. An 8.0 μm pore membrane was inserted to allow the cells to migrate, and the plate was incubated at 37°C for 24 h. The migrated cells were fixed, stained with crystal violet, and ten random fields of each well were counted.

### Cell apoptosis and cell cycle assays

Cell apoptosis in transfected cells was analyzed using a FITC-Annexin V apoptosis kit (Yeasen, Shanghai). In brief, cultured cells were suspended, centrifuged, washed with PBS, and resuspended in cold Binding Buffer. FITC-Annexin V reagent (1.25 μL) was added to the cells and apoptotic cells were assessed using a A00-1-1122 flow cytometer (Beckman Kurt Biotechnology (Suzhou) Co., Ltd).

For cell cycle analysis, the transfected cells were fixed with 70% cold ethanol (700 μL) at 4°C for 4 h, washed three times with PBS, and stained with the fluorescent dye propidium iodide (PI; Sigma) at 37°C for 30 min in the dark. The ratio of cells in various cell cycle phases was assessed using a Beckman flow cytometer.

### Measurement of caspase 3/7 activity

In brief, a total of 10^4^ siCtrl, si*MARCH9*#1 and si*MARCH9*#2 HCT116 and RKO cells were seeded into 96-well plates. 1.5 hours later, the activity of caspase 3/7 was measured by using Caspase-Glo reagent (Promega), according to the manufacturers’ protocols.

### Xenograft model assay

Female BALB/c nude mice that were 6 weeks old (Beijing vital-river Co.td) were raised in a pathogen-free facility and divided into a sh*MARCH9* group (n=5) and a shCtrl group (n=5). The mice were subcutaneously injected with sh*MARCH9*#1 or shCtrl transfected HCT116 cells (4 × 10^6^ cells) in the right axilla. After 45 days, the mice were anesthetized and sacrificed, and the tumors were excised, weighed, and photographed. Tumor tissues were used to detect the expression of MARCH9 by western blotting.

### Statistical analysis

All the experimental results was used to analyze in GraphPad Prism 7 software and data were expressed as the mean ± SD. The Student’s t-test was used to compare differences between the groups. Statistical significance was considered at *p* < 0.05.

## Results

### MARCH9 was highly expressed in CRC tissue

**Figure 1:**
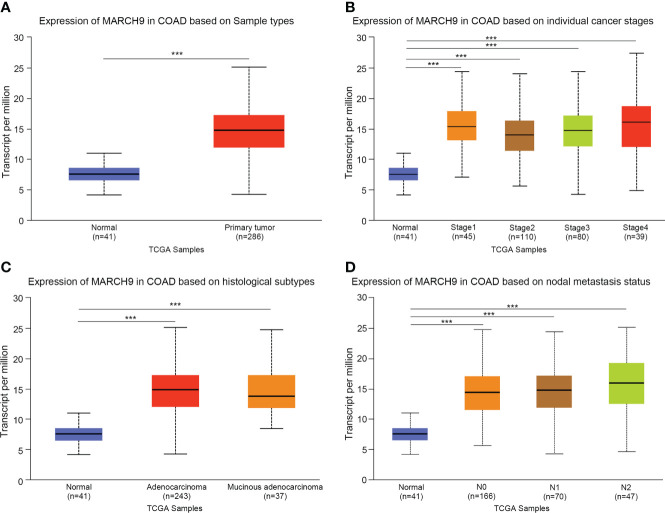
MARCH9 was highly expressed in CRC tissue. **(A)** Expression of MARCH9 in tumors vs normal controls. **(B)** Expression of MARCH9 in different cancer stages. **(C)** Expression of MARCH9 in different histological subtypes. **(D)** Expression of MARCH9 in nodal metastasis ***p<0.001.

We firstly explored the clinical significance of MARCH9 in CRC. Based on TCGA data, MARCH9 expression was significantly higher in CRC than adjacent normal tissue samples (**Figure 1A**). MARCH9 expression was also much higher in CRC stages 1-4 (**Figure 1B**), adenocarcinoma and mucinous adenocarcinoma (**Figure 1C**), and N0-N2 samples (**Figure 1D**).

### Knockdown of *MARCH9* inhibited CRC cell proliferation and migration

**Figure 2:**
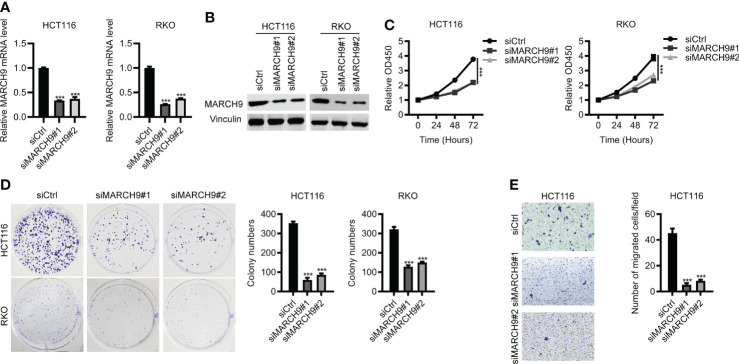
Knockdown of *MARCH9* inhibited CRC cell proliferation and migration. **(A, B)** RT-qPCR and Western blot results of MARCH9 mRNA and protein levels in two CRC cell lines transfected with two *MARCH9* siRNAs for 48 (h) **(C)** CCK-8 assay results of cell viability in two CRC cell lines transfected with two *MARCH9* siRNAs at 24, 48, and 72h. **(D)** Cell colony formation results of two CRC cell lines transfected with two *MARCH9* siRNAs at 14 days. **(E)** Transwell assay results of cell migration ability in two CRC cell lines transfected with two *MARCH9* siRNAs at 48 (h) ***P < 0.001.

Next, we investigated the role of MARCH9 in CRC by using loss-of-function assay. As shown by RT-qPCR and Western blot analysis, both si*MARCH9*#1 and si*MARCH9*#2 could effectively knock down MARCH9 expression in two CRC cell lines (**Figures 2A, B**). The CCK-8 assay was used to investigate whether MARCH9 knockdown influenced CRC cell proliferation. CRC cell lines transfected with si*MARCH9*#1 and si*MARCH9*#2 had significantly lower viability than the siCtrl group (**Figure 2C**). Knockdown of *MARCH9* also inhibited the colony formation and migration of CRC cells (**Figures 2D, E**). We also checked the effect of MARCH9 on the proliferation of colorectal normal cells. The results showed that knockdown of *MARCH9* suppressed the proliferation of FHC cells (**Supplementary Figure 1**). Therefore, MARCH9 expression is critical for the proliferation and growth of colorectal normal and CRC cells.

### Overexpression of *MARCH9* promoted CRC cell proliferation and migration

**Figure 3:**
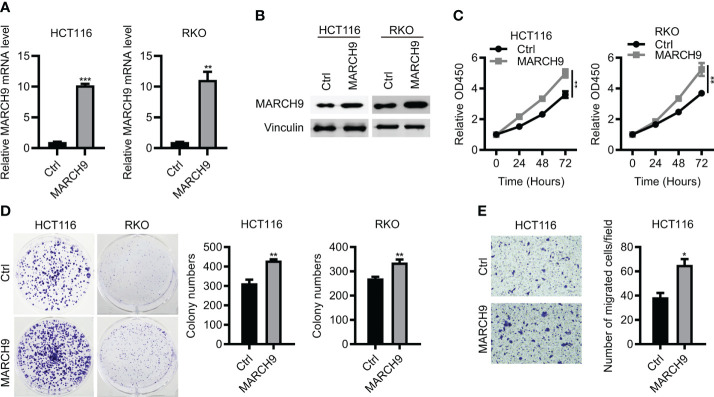
Overexpression of *MARCH9* promoted CRC cell proliferation and migration. **(A, B)** RT-qPCR and western blot results of MARCH9 mRNA and protein levels in CRC cell lines transfected with *MARCH9* or Ctrl plasmids at 48 (h) **(C)** CCK-8 assay results of cell viability in two CRC cell lines transfected with *MARCH9* plasmids and Ctrl at 24, 48, and 72h. **(D)** Cell colony formation results of two CRC cell lines transfected with MARCH9 plasmids and Ctrl at 14 days. **(E)** The migration ability of two CRC cell lines transfected with *MARCH9* plasmids and Ctrl at 48 **(h)** *P < 0.05; **P < 0.01; ***P < 0.001.

To confirm the function of MARC9 in CRC, we then performed gain-of-function experiments. As shown by RT-qPCR and Western blot analysis, endogenous MARCH9 expression was greatly upregulated after *MARCH9* overexpression in two CRC cell lines (**Figures 3A, B**). This resulted in an increase in cell viability and proliferation (**Figures 3C, D**). MARCH9 overexpression also enhanced CRC cell migration, supporting an oncogenic role for this protein in CRC. These results confirmed the oncogenic function of MARCH9 in CRC (**Figure 3E**).

### MARCH9 regulated CRC cell apoptosis and cell cycle

**Figure 4:**
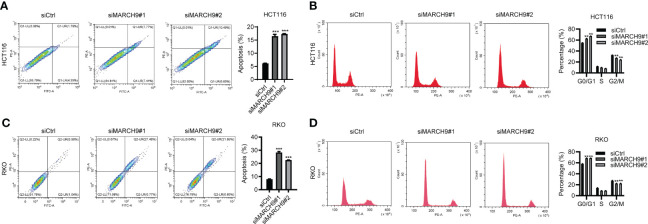
MARCH9 regulated CRC cell apoptosis and cell cycle. **(A, B)** The cell apoptosis rate of HCT116 and RKO cells after *MARCH9* knockdown at 48 (h) **(C, D)** The cell cycle distribution of HCT116 and RKO cells after *MARCH9* knockdown at 48 (h) **P < 0.01; ***P < 0.001.

We then explore the role of MARCH9 in CRC cell apoptosis and cell cycle progression. *MARCH9* knockdown significantly promoted apoptosis in HCT116 and RKO cells as shown by flow cytometry (**Figures 4A, B**). Furthermore, downregulation of MARCH9 enhanced the caspase 3/7 activity in both cell lines (**Figure S2**), suggesting the underlying mechanisms how MARCH9 regulates apoptosis. There was a higher number of cells in the G0/G1 phase of the cell cycle and a lower number in the S and G2/M phases in two CRC cell lines (**Figures 4C, D**). These results indicated that MARCH9 downregulation induced cell apoptosis and blocked the cell cycle in the G0/G1 phase.

### MARCH9 downregulated CYLD and activated P65

**Figure 5:**
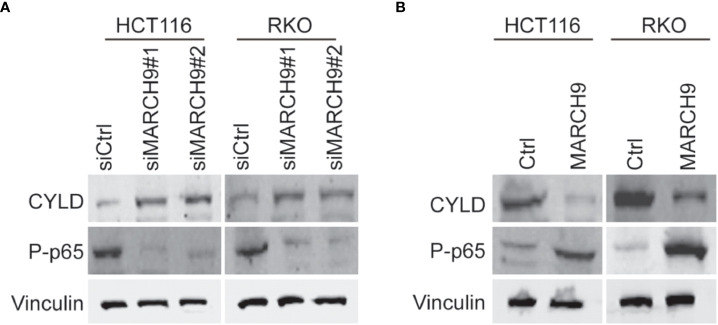
MARCH9 downregulated CYLD and activates P65. **(A)** Expression of CYLD, p65 and P-p65 in two CRC cell lines after *MARCH9* knockdown by western blotting. **(B)** The expression of CYLD, p65 and P-p65 in two CRC cell lines following *MARCH9* overexpression by western blotting.

Cylindromatosis (*CYLD*) is an important tumor suppressor gene that inhibits the development of various cancers through inactivation of NFκB (p65) (25, 26). A wide range of studies have shown that the expression of CYLD could be suppressed at transcription level by different non-coding RNAs (26–28), whereas whether CYLD abundance was repressed at post-transcription manner in colorectal cancer should be determined. Based on western blot results, we showed that knockdown of *MARCH9* induced CYLD protein expression and reduced p65 phosphorylation in two CRC cell lines (**Figure 5A**), while overexpression of MARCH9 had the opposite effect on CYLD expression and p65 phosphorylation (**Figure 5B**). Total protein expression of p65 did not change after knocking down or overexpressing *MARCH9* (**Figure 5**), indicating that MARCH9 regulates p65 phosphorylation but not expression levels. These results suggest that MARCH9 suppresses the expression of CYLD at post-transcription manner, leading to activation of p65.

### MARCH9 accelerated CRC progression by promoting p65

**Figure 6:**
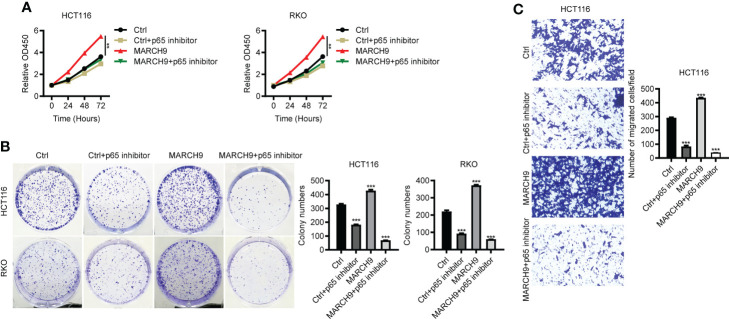
MARCH9 accelerated CRC progression by promoting p65. **(A)** The cell viability of two CRC cell lines transfected with Ctrl plasmids, Ctrl plasmids+p65 inhibitor, *MARCH9* plasmids and *MARCH9* plasmids+p65 inhibitor at 24, 48, and 72h using a CCK-8 assay. **(B)** Cell colony formation results of two CRC cell lines transfected with Ctrl plasmids, Ctrl plasmids+p65 inhibitor, *MARCH9* plasmids and *MARCH9* plasmids+p65 inhibitor at 14 days. **(C)** The migration ability of HCT116 and RKO cells transfected with Ctrl plasmids, Ctrl plasmids+p65 inhibitor, *MARCH9* plasmids and *MARCH9* plasmids+p65 inhibitor at 48 (h) **P < 0.01; ***P < 0.001.

To explore the role of p65 activity regulated by MARCH9 in CRC, we treated Ctrl and *MARCH9* CRC cells with a specific p65 inhibitor, named Caffeic Acid Phenethyl Ester (CAFE). While *MARCH9* overexpression promoted CRC cell viability (**Figure 6A**), increased the number of cell colonies (**Figure 6B**), and increased cell migration (**Figure 6C**), co-expression of CAFE weakened these tumor-promoting effects. By contrast, the inhibitory effect of CAFÉ on the growth and migration of Ctrl cells was smaller than that of *MARCH9* overexpressing cells (**Figures 6A-C**). These results suggest that MARCH9 may accelerate CRC progression by regulating p65.

### *MARCH9* knockdown suppressed tumor growth *in vivo*

**Figure 7:**
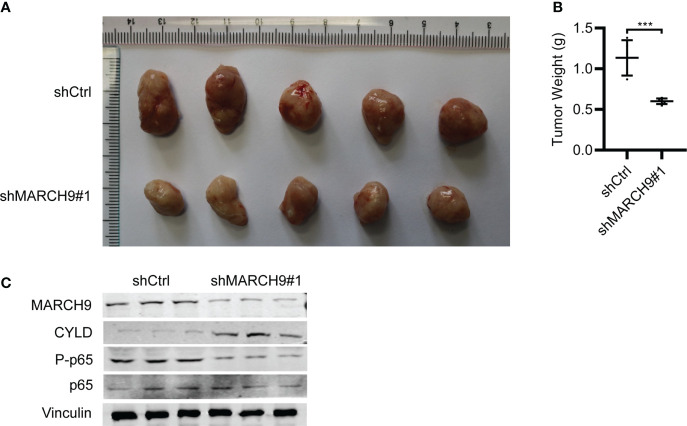
*MARCH9* knockdown suppressed tumor growth *in vivo*. **(A)** Xenograft tumors were excised from BALB/c nude mice on day 45 and compared between sh*MARCH9*#1 and shCtrl groups. **(B)** Tumor weight of BALB/c nude mice between sh*MARCH9*#1 and shCtrl groups in statistics. **(C)** MARCH9, CYLD, P-p65 and p65 expression in xenograft tumor tissues between sh*MARCH9*#1and shCtrl groups by western blotting. ***P < 0.001.

Lastly, we studied the *in vivo* function of MARCH9 by implanting shCtrl and shMARCH9 CRC into the BALB/C nude mice. The sh*MARCH9#*1 group of BALB/C nude mice had smaller tumors with significantly lower weight than the shCtrl group (**Figures 7A, B**). Western blot analysis revealed lower expression of MARCH9 and higher expression of CYLD in the xenograft tumor tissues from the sh*MARCH9#*1 group than tissues from the siCtrl group (**Figure 7C**). In addition, *MARCH9* knockdown suppressed the phosphorylation of p65, while had no effect on the total protein expression of p65 in the tumor tissues (**Figure 7C**). Collectively, these data suggest that *MARCH9* knockdown suppressed HCT116 cell tumorigenicity.

## Discussion

Ubiquitination and deubiquitination are reversible post-translational modification processes that regulate both tumor-suppressing and -promoting pathways (29). MARCH9 is a member of the MARCH ubiquitin ligase family that targets cell surface receptors for degradation (22). MARCH9 plays a pivotal role in antigen-presenting cells by regulating the protein stability of human leukocyte antigen (HLA)-DM (30). In addition, MARCH9 modulates the export of major histocompatibility complex class I (MHC I) based on the regulation of MARCH9 on MHC I’s ubiquitination (31). These results indicate the significance of MARCH9 in immune system. Despite these studies, the clinical significance and the precise role of MARCH9 during the development of malignant tumors, including CRC remain to be determined. In this study, we explored the role of MARCH9 on CRC progression both *in vitro* and *in vivo*. MARCH9 expression was significantly upregulated in CRC samples from the TCGA database by individual cancer stage, histological subtype, and nodal metastasis status. While knockdown of MARCH9 inhibited CRC cell growth and migration, overexpression of *MARCH9* had the opposite effect. *In vivo* xenograft studies confirmed that *MARCH9* knockdown suppressed tumor growth, resulting in smaller and lower weight tumors than observed in the control group. Knockdown of *MARCH9* also promoted CRC cell apoptosis and caused cell cycle arrest, indicating that MARCH9 functions as an oncogene during this disease. Taken together, MARCH9 is a promising diagnosis biomarker and therapeutic target for CRC.

Previous studies have shown that MARCH9 targets a wide range of downstream substrates involved in immune system, whereas the substrates of MARCH9 in cancer development are less clear. Identifying the molecular mechanisms contributing to the MARCH9-mediated CRC progression will help us develop effective drugs to treat the CRC patients with highly expressed MARCH9. Based on Western blots, we showed that MARCH9 could downregulate CYLD expression and activate p65 expression. CYLD is a K63-specific deubiquitinase that functions as a tumor suppressor in many cancers (32). Yang et al. reported that CYLD could inhibit growth and promote apoptosis of CRC cells (26). This is consistent with the results of the current study. In addition, CYLD disrupts key protein-protein interactions that are important for the activation of the Nuclear Factor-κB (NF-κB) pathway (33). NF-κB plays an important role in cancer-related processes and regulates cell proliferation, apoptosis, angiogenesis, and metastasis during CRC (34). p65 is one of the five known members of the NF-κB protein family, which form hetero or homodimers and bind to inhibitory proteins (35). Findings from the current study showed that CYLD expression was upregulated and p65 was downregulated in HCT116 and RKO cells after MARCH9 knockdown. Conversely, CYLD expression was downregulated and p65 was upregulated following *MARCH9* overexpression. These results indicate that MARCH9 regulated CYLD and p65 expression in CRC cells. Importantly, cell viability, colony formation, and migration assays confirmed that MARCH9 overexpression conferred higher sensitivity of CRC cells to the treatment of p65 inhibitors, suggesting that MARCH9 promoted CRC progression by activating p65. These results also implied that p65 inhibitors might be a therapeutic strategy for the treatment of CRC patients with overexpression of MARCH9.

In summary, our results confirmed that MARCH9 was overexpressed in CRC patients and its overexpression contributed to the proliferation, migration and tumorigenesis of CRC cells, suggesting that it may function as an oncogene during this disease. The findings also illustrated that MARCH9 could serve as a very promising biomarker for CRC therapy. Importantly, MARCH9 activated p65 signaling and inhibition of p65 completely reversed the oncogenic function of MARCH9, implying that p65 could be the therapeutic target for MARCH9 overexpressed CRC patients. However, further clinical trials are needed to confirm these results.

## Data availability statement

The datasets presented in this study can be found in online repositories. The names of the repository/repositories and accession number(s) can be found in the article/**Supplementary Material**.

## Ethics statement

The animal study was reviewed and approved by Animal Care and Use Committee of the Minzu University of China (ECMUC2019001AO).

## Author contributions

YC and LC conceived and designed this study. BC and HL carried out the experiment and analyzed the experiment data. HL, BC, and L-LL drafted the manuscript with critical revisions from YC and LC. All authors critically reviewed the manuscript.

## Conflict of interest

The authors declare that the research was conducted in the absence of any commercial or financial relationships that could be construed as a potential conflict of interest.

## Publisher’s note

All claims expressed in this article are solely those of the authors and do not necessarily represent those of their affiliated organizations, or those of the publisher, the editors and the reviewers. Any product that may be evaluated in this article, or claim that may be made by its manufacturer, is not guaranteed or endorsed by the publisher.
