# Supplementary material for: The role of MARCH9 in colorectal cancer progression
Source: Front Oncol. 2022 Sep 16;12:906897. doi: 10.3389/fonc.2022.906897 (PMC9523723; doi:10.3389/fonc.2022.906897)
Supplement: Supplementary file 1 [file DataSheet_1.docx]

Figure S1. The effect of MARCH9 knockdown on the proliferation of FHC cells.


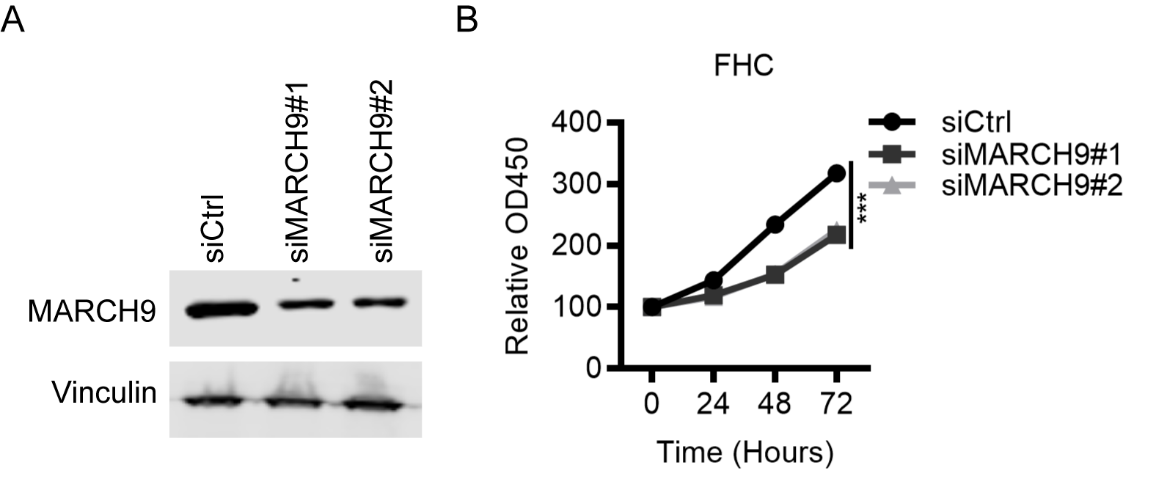


(A) Western blot results of MARCH9 protein levels in FHC cell lines transfected with two MARCH9 siRNAs for 48 h. (C) CCK-8 assay results of cell viability in FHC cell lines transfected with two MARCH9 siRNAs at 24, 48 and 72h.

Figure S2. MARCH9 knockdown enhances caspase 3/7 activity in CRC cells.


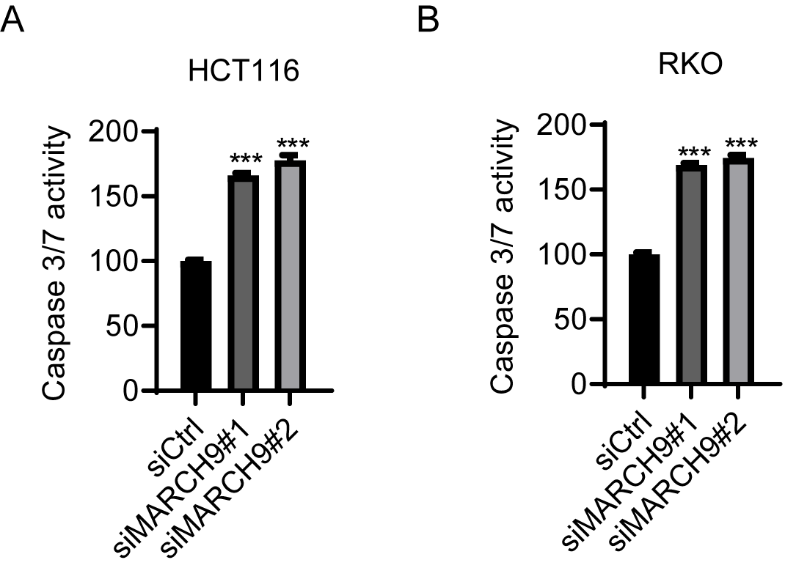


(A and B) Caspase 3/7 activity was measured in HCT116 and RKO cell lines transfected with two MARCH9 siRNAs ***p<0.001.
